# Alcohol and head and neck cancer risk in a prospective study

**DOI:** 10.1038/sj.bjc.6603713

**Published:** 2007-03-27

**Authors:** N D Freedman, A Schatzkin, M F Leitzmann, A R Hollenbeck, C C Abnet

**Affiliations:** 1 Cancer Prevention Fellowship Program, Division of Cancer Prevention, National Cancer Institute, National Institutes of Health, Executive Plaza North, Suite 3109, 6130 Executive Boulevard, MSC 7361, Bethesda, MD 20892-7361, USA; 2 Nutritional Epidemiology Branch, Division of Cancer Epidemiology and Genetics, National Cancer Institute, NIH, 6120 Executive Blvd, EPS/320, MSC 7232, Rockville, MD 20852, USA; 3 AARP, 601 E Street NW, Washington, DC 20049, USA

**Keywords:** head and neck cancer, alcohol, cohort

## Abstract

We investigated the relation between head and neck cancer risk and alcohol consumption in the NIH-AARP Diet and Health Study. During 2 203 500 person-years of follow-up, 611 men and 183 women developed head and neck cancer. With moderate drinking (up to one alcoholic drink per day) as the referent group, non-drinkers showed an increased risk of head and neck cancer (men: hazard ratio (HR) 1.68, 95% confidence interval (95% CI) 1.37–2.06; women: 1.46, 1.02–2.08). Among male and female alcohol drinkers, we observed a significant dose–response relationship between alcohol consumption and risk. The HR for consuming >3 drinks per day was significantly higher in women (2.52, 1.46–4.35) than in men (1.48, 1.15–1.90; *P* for interaction=0.0036). The incidence rates per 100 000 person-years for those who consumed >3 drinks per day were similar in men (77.6) and women (75.3). The higher HRs observed in women resulted from lower incidence rates in the referent group: women (14.7), men (34.4). In summary, drinking >3 alcoholic beverages per day was associated with increased risk in men and women, but consumption of up to one drink per day may be associated with reduced risk relative to non-drinking.

Each year, more than 600 000 individuals are diagnosed with squamous cancers of the head and neck worldwide (Parkin *et al*, 2005). Head and neck cancer has multiple anatomic sub-sites, including the oral cavity, pharynx, and larynx, which may have distinct aetiologies.

Alcohol use has consistently been indicated as a risk factor for head and neck cancer (Sturgis *et al*, 2004). However, owing to small case numbers, previous prospective studies have typically collapsed cancers of the head and neck and the aesophagus into a single broad category of upper-gastrointestinal tract sites (Gronbaek *et al*, 1998; Kjaerheim *et al*, 1998; Boeing, 2002). Few prospective studies have examined the association between alcohol and individual head and neck cancer sub-sites, such as the larynx, oral cavity, or pharynx (Schottenfeld *et al*, 1974; Boffetta and Garfinkel, 1990; Adami *et al*, 1992; Tonnesen *et al*, 1994; Sigvardsson *et al*, 1996; Boffetta *et al*, 2001). Most of these studies have not adjusted for tobacco use, an important potential confounder of the relation between alcohol and head and neck cancer (Boffetta and Garfinkel, 1990). Also, few studies (Franceschi *et al*, 1994; Gronbaek *et al*, 1998; Hayes *et al*, 1999; Castellsague *et al*, 2004; Polesel *et al*, 2005) have examined non-drinkers and those that drink less than two drinks per day separately, an important comparison as consumption of less than two drinks per day may reduce cardiovascular disease risk (Maclure, 1993).

Although men are 2–7 times more likely to develop these cancers than women (depending on sub-site) (Parkin *et al*, 2005), few epidemiological studies have compared the association of alcohol and head and neck cancer in men to that in women (Blot *et al*, 1988; Franceschi *et al*, 1994; Hayes *et al*, 1999; Boffetta *et al*, 2001). These studies generally indicate higher relative risks in women than in men, although many had small case numbers and all but one were case–control studies precluding the examination of incidence rates.

Alcohol consumption is increasing in many countries worldwide, particularly among women (Boffetta and Hashibe, 2006), and this highlights the need to better understand the association between alcohol consumption and head and neck cancer. We prospectively investigated this association in 492 960 participants of the NIH-AARP diet and health study.

## MATERIALS AND METHODS

The establishment and recruitment procedures of the NIH-AARP Diet and Health Study have been described (Schatzkin *et al*, 2001). Between 1995 and 1996, a dietary questionnaire was mailed to 3.5 million members of AARP, formerly known as the American Association of Retired Persons, a large US organisation whose membership is open to those 50 years of age or older. These members resided in one of eight US states (California, Florida, Louisiana, New Jersey, North Carolina, Pennsylvania, Georgia, or Michigan). A total of 566 407 respondents (308 692 men and 211 702 women) sufficiently completed the survey and consented to participate in the study. We excluded proxy respondents (*n*=15 760) and subjects with cancer at baseline (*n*=51 219), calorie intake more than two interquartile ranges from the mean (*n*=4419), who died or were diagnosed with cancer on the first day of follow-up (*n*=10), and who failed to provide information on alcohol use (*n*=2039). The resulting analytical cohort comprised 492 960 participants.

### Cohort follow-up

Addresses for members of the NIH-AARP cohort were updated annually by matching the cohort database to that of the USA Postal Service change of address database (Michaud *et al*, 2005). Vital status was ascertained by annual linkage to the USA Social Security Administration death master file and responses to mailings. Follow-up time extended from study baseline (between 1995 and 1996) until diagnosis of a head and neck, aesophageal, or gastric cancer (as diagnosis of one of these cancers is associated with increased surveillance of the other sites), date of death, end of study (31 December 2000), or the date moved out of registry ascertainment area.

Incident cases of cancer were identified by probabilistic linkage between the NIH-AARP cohort membership and eight state cancer registry databases. We estimate that 90% of cancer cases are detected in the cohort by this approach (Michaud *et al*, 2005). Cancer sites were identified by anatomic site and histologic code of the International Classification of Disease for Oncology, third edition (Fritz, 2000). All cases of head and neck cancer with squamous histology were considered for this analysis. We classified tumours with site codes C32.0–C32.9 as laryngeal cancer. Oral cavity cancers included tumours with codes C00.1–C06.9. Cancers of the oropharynx and hypopharynx included tumours of the tonsil (C09.0–C09.9), oropharynx (C10.0–C10.9), pyriform sinus (C12.9), hypopharynx (C13.0–C13.9), and pharynx not otherwise specified (NOS) (C14.0). The overarching head and neck cancer category included those diagnosed with a cancer of the oral cavity, oropharynx and hypopharynx, larynx, and those with other squamous tumours in the head or neck or overlapping region of the lip, oral cavity, and pharynx.

### Exposure assessment

The baseline questionnaire contained questions about demographics, daily alcohol, cigarette use, physical activity, and a 124-item food frequency questionnaire (Schatzkin *et al*, 2001). Usual daily alcohol (wine, beer, and liquor), fruit, and vegetable intakes were calculated from the questionnaire as pyramid servings as defined by the USA Department of Agriculture (USDA), taking account of intake frequency and serving size (Subar *et al*, 2000; Schatzkin *et al*, 2001). One drink corresponds to one serving of the USDA food guide pyramid: one 12 fluid ounce beer, one 5 fluid ounce glass of wine, or one 1.5 ounce shot of liquor (each approximately 13 g of alcohol). We used categorical variables of alcohol intake: none, up to 1 drink per day, 1–3 drinks per day, and >3 drinks per day. Self-administered questionnaires for alcohol intake have high reproducibility (0.84 after 4 years) and validity with respect to 1 week diet records (0.92 for men and 0.90 for women) (Giovannucci *et al*, 1991).

To adjust for smoking, we used smoking status (current, former, and never) and usual smoking dose to construct a categorical variable (smoke-quit-dose): never smokers, former smokers who smoked less than one pack per day, former smokers who smoked more than one pack per day, current smokers who smoked less than one pack per day, and current smokers who smoked more than one pack per day. We lacked information on smoking duration.

### Statistical analysis

Analyses were performed with SAS version 8.2. Potential confounders were tabulated by alcohol intake. Hazard ratios (HR) and 95% confidence intervals (95% CI) were calculated using Cox proportional hazards regression (Cox, 1972). We tested the proportional hazards assumption by modelling interaction terms of time and categories of alcohol intake and found no statistically significant interactions. Follow-up time was used as the underlying time metric. Using age as the underlying time metric did not affect results. All multivariate models were adjusted by categorical variables for education, body mass index (World Health Organization categories), smoke-quit-dose, vigorous physical activity, activity throughout the day, and continuous measures for vegetable intake, fruit intake, total energy intake, and age at baseline. Owing to significant differences in the risk of non-drinkers relative to the risk of moderate alcohol drinkers of up to one drink per day, we used moderate drinkers as the referent category. The test for trend was restricted to alcohol drinkers and used a linear variable where each subject was assigned the median intake value for their category. Models that analysed categories of liquor, beer, or wine consumption further included the other two types of alcohol as categorical variables, to account for total alcohol consumption in each model. Small numbers of subjects had missing data values for some covariates. Missing covariates were represented by dummy variables in the models. Excluding those with missing covariate information slightly reduced case numbers but did not materially change point estimates. An alpha level of less than 0.05 was considered statistically significant and all tests were two sided. We tested for an interaction between categorical variables of alcohol intake and gender by using cross-product terms with a three degree of freedom (df) likelihood ratio *χ*^2^ test. Age-adjusted incidence rates were calculated as in Breslow and Day (1987) with 5-year age bands and age- and gender-specific rates standardised to the entire NIH-AARP Diet and Health study population.

## RESULTS

As shown in Table 1, alcohol drinkers were more likely to smoke and to have received more formal education than non-drinkers, but they consumed less fruit on average. The distribution of alcohol use within the AARP cohort, 24.1% (119 007) non-drinkers, 53.1% (261 889) moderate drinkers of 0–1 alcoholic beverages per day, 15.2% (74 854) drinkers of 1–3 alcoholic beverages per day, and 7.5% (37 210) drinkers of >3 drinks per day, was similar to that in the United States overall, as data from the 1999–2000 NHANES survey indicated 35.5% non-drinkers, 42.7% moderate drinkers of 0–1 alcoholic beverages per day, 17.9% drinkers of 1–3 alcoholic beverages per day, and 4.0% drinkers of ⩾3 alcoholic beverages per day (Breslow *et al*, 2006).

After 2 203 500 person-years of follow-up, 794 participants were diagnosed with head and neck cancer. We found that gender significantly modified the relation between alcohol and head and neck cancers (*P*=0.0036, 3 df) and present gender stratified estimates throughout. The age-standardised incidence rate per 100 000 person-years among moderate drinking women (up to one drink per day) (14.7, 95% CI 11.3–18.0) was lower than moderate drinking (34.4, 30.0–39.0) men. Among drinkers, increasing alcohol consumption was significantly associated with increased risk. The incidence rates in those who drank >3 alcoholic drinks per day were similar in men and women (women: 75.3, 40.5–110.1; men: 77.6, 63.1–92.1) (Table 2). In age-adjusted models (Table 2), men and women who drank >3 alcoholic beverages per day were at increased risk relative to moderate drinkers (men: 2.25, 1.79–2.82; women: 5.16, 3.08–8.63). The association between increased alcohol consumption and risk among drinkers of alcohol was attenuated but remained statistically significant in multivariate adjusted models. Men (1.48, 1.15–1.90) and women (2.52, 1.46–4.35) who drank >3 drinks per day were at increased risk relative to moderate drinkers (Table 3).

When examined by alcohol type, consuming more than three drinks of liquor or beer per day was associated with increased risk relative to moderate consumption of these beverages in both men and women (Table 3).

We also investigated the association between alcohol intake and head and neck cancer sub-sites. Women who drank more than three alcoholic drinks per day were at significantly increased risk of oral cavity cancer (2.81, 1.29–6.11) and nonsignificantly increased risk of oropharyngeal and hypopharyngeal cancer (1.97, 0.42–9.31) and laryngeal cancer (2.15, 0.82–5.65) relative to moderate alcohol drinkers. Men who drank more than three alcoholic drinks per day were at significantly increased risk for cancers of the oral cavity (1.52, 1.01–2.27), oropharynx and hypopharynx (2.32, 1.29–4.18), but not the larynx compared to moderate drinkers (1.37, 0.91–2.05) (Table 4).

In both age-adjusted models (Table 2; men: 1.77, 1.45–2.16; women: 1.39, 0.98–1.98) and multivariate adjusted models (Table 3; men: 1.68, 1.37–2.06; women: 1.46, 1.02–2.08), non-drinkers were at increased risk of head and neck cancer relative to moderate alcohol drinkers. When examined by alcohol type (Table 3), non-drinking men were at increased risk relative to moderate drinkers of wine (1.34, 1.10–1.63), beer (1.18, 0.96–1.45), and liquor (1.28, 1.03–1.58), whereas non-drinking women were at increased risk relative to moderate drinkers of wine (1.58, 1.12–2.25) and liquor (1.16, 0.80–1.66).

We performed several sensitivity analyses. Years of education and alcohol intake were positively correlated (Pearson correlation of 0.12); so we stratified our analysis on years of education. The stratified estimates for non-drinkers relative to moderate drinkers were 1.70 (1.21–2.37) for men and 2.57 (1.20–5.53) for women who had a college education or greater and 1.76 (1.35–2.27) for men and 1.35 (0.89–2.03) for women without a college education. We also stratified by smoking status (current, former, and never). Non-drinking men and women were at elevated risk relative to moderate drinkers among current (men: 2.00, 1.39–2.88; women: 1.28, 0.77–2.13), former (men: 1.58, 1.17–2.13; women: 1.77, 0.94–3.34), and never smokers (men: 1.35, 0.85–2.15; women: 1.53, 0.59–3.94). We also excluded those who reported poor health on the questionnaire. After excluding 34 580 men and 25 013 women who reported poor health at baseline, non-drinking men (1.73, 1.32–2.17) and non-drinking women (1.67, 1.12–2.51) were at increased risk relative to moderate drinkers. Similarly, after excluding the first 2 years of follow-up, non-drinking participants remained at increased risk (men: 1.59, 1.21–2.09; women: 1.35, 0.94–4.25).

## DISCUSSION

In a large prospective study, we investigated the association between alcohol and head and neck cancer in men and women separately. Consistent with previous studies (Blot *et al*, 1988; Boffetta and Garfinkel, 1990; Adami *et al*, 1992; Franceschi *et al*, 1994; Gronbaek *et al*, 1998; Kjaerheim *et al*, 1998; Hayes *et al*, 1999; Boffetta *et al*, 2001; Boeing, 2002; Castellsague *et al*, 2004; Sturgis *et al*, 2004; Polesel *et al*, 2005; Boffetta and Hashibe, 2006), predominantly of men, we observed a significant dose–response association between increasing alcohol consumption and head and neck cancer in both male and female alcohol drinkers. Results were generally similar for beer, liquor, and wine, although only 0.3% (1528) of the cohort (1064 men and 464 women) drank >3 glasses of wine per day limiting power. Hazard ratios for head and neck cancer sub-sites were also generally similar.

In this study, although the absolute age-standardised incidence rates among those who drank 1–3 alcoholic drinks per day and those who drank >3 alcoholic drinks per day were similar in men and women, the HR for the association between alcohol intake and head and neck cancer risk were significantly higher for women than for men. Similar increased risks among women were observed in most previous studies of this association (Blot *et al*, 1988; Franceschi *et al*, 1994; Hayes *et al*, 1999; Boffetta *et al*, 2001). In our study, the higher HR observed in women compared to men result from lower incidence rates in the referent group. These differences in baseline incidence suggest the presence of additional gender-specific risk or protective factors. These factors might include occupational carcinogens (Boffetta *et al*, 2003) or reproductive hormones (Yang *et al*, 1989; Olsson *et al*, 2003).

Compared to not-drinking, drinking up to one alcoholic drink per day was associated with reduced head and neck cancer risk. Although some previous epidemiological studies have observed a protective association between moderate alcohol consumption and cancers of the head and neck and the aesophagus, results overall have been inconsistent (Franceschi *et al*, 1994; Gronbaek *et al*, 1998; Hayes *et al*, 1999; Castellsague *et al*, 2004; Polesel *et al*, 2005). Most previous studies were conducted in populations that drink larger amounts of alcohol (Altieri *et al*, 2005) than that of the current study.

The observed inverse association could reflect exposure misclassification if former heavy drinkers had stopped drinking at the time of the questionnaire. Excluding either those subjects who reported poor health or the first 2 years of follow-up did not appreciably alter risk estimates and alcohol consumption was assessed before cancer diagnosis. However, some studies suggest that former drinkers remain at elevated risk for head and neck cancer, even 10 years after use (Hayes *et al*, 1999; Castellsague *et al*, 2004). Because we lacked information on past alcohol use, misclassification of non-drinkers may have affected our risk estimates.

It is also possible that moderate alcohol drinking is a proxy for a healthy or privileged lifestyle or an associated exposure. We did adjust models for head and neck cancer risk factors as well as markers of lifestyle, including age, education, body mass index, smoking, physical activity, fruit intake, vegetable intake, and total energy intake. However, the observed protective association could still be due to an unmeasured or poorly measured confounder in our analysis, such as socioeconomic status (SES). Years of education, as with the other covariates used for adjustment, did not attenuate the protective association. We additionally explored SES as a confounder using median income of the census tract in which the participant lived. Inclusion of this variable in the multivariate analysis did not alter risk estimates (data shown). We also examined possible effect modification by smoking status, as health conscious individuals may be more likely to drink moderate quantities of alcohol and less likely to smoke. Although the risk estimates for moderate alcohol consumption were similar among current, former, and never smokers, few cases with head and neck cancer were non-smokers (95 men and 20 women), limiting the power to investigate this stratum.

This study has a number of strengths. It is a large prospective analysis with exposure information collected before cancer diagnosis. We present models separately for men and women and we examined the associations for both total head and neck cancer and by sub-sites. To limit confounding, we adjusted our models for most of the major risk factors for head and neck cancer, including cigarette smoking, a strong risk factor that is associated with alcohol use. Furthermore, the large number of participants who consumed less than one alcoholic drink per day allowed us to investigate the association between moderate drinking and risk. This study also had several limitations. We lacked information on participant income and occupation, factors that could confound the association between alcohol intake and risk. We also lacked information about past alcohol consumption and smoking duration.

In conclusion, among alcohol drinkers, increasing consumption of alcohol was associated with increased risk of head and neck cancer in both men and women. Although consumption of >3 drinks per day had a bigger HR for head and neck cancer in women than in men, incidence rates for men and women in this category were similar. Men and women who reported consuming less than one drink per day had lower rates of head and neck cancer than men and women who reported no alcohol consumption. The observed association between increasing alcohol consumption and increased head and neck cancer in men and women is consistent with many previous studies. In contrast, the observed protective association with drinking up to one alcoholic beverage per day should be interpreted cautiously and must be investigated further, particularly in prospective studies that collect information on lifetime use of alcohol.

## Figures and Tables

**Table 1:** Study characteristics by alcohol intake

	**Alcohol intake (drinks per day)^a^**							
	**Zero**		**<1**		**1–3**		**>3**	
	**Number**	**%**	**Number**	**%**	**Number**	**%**	**number**	**%**
Entire cohort	119 007	24.1	261 889	53.1	74 854	15.2	37 210	7.5
*Gender*								
Male	60 761	20.7	147 104	50.0	54 584	18.6	31 851	10.8
Female	58 246	29.3	114 785	57.8	20 270	10.2	5359	2.7
Age (years)^b^	63.0	58.2–66.8	62.3	57.4–66.4	62.9	58.1–66.8	62.7	57.9–66.5
Body mass index (kg m^−2^)^b^	26.7	24.1–30.4	26.5	23.9–29.4	25.8	23.6–28.3	26.5	24.2–29.1
Total daily calories^b^	1616.7	1206.8–2155.1	1606.8	1223.9–2092.8	1802.8	1421.7–2283.3	2397.8	1899.7–3095.1
Vegetable consumption (servings per day)^b^	3.3	2.1–4.9	3.4	2.3–4.9	3.7	2.5–5.2	3.6	2.4–5.2
Fruit consumption (servings per day)^b^	2.5	1.4–4.1	2.5	1.5–3.8	2.4	1.4–3.6	1.9	1.0–3.2
								
*Education (%)*								
Less than high school	11 522	39.5	12 995	44.5	2770	9.5	1897	6.5
12 years (completed high school)	30 055	31.2	49 582	51.5	10 571	11.0	6039	6.3
Some post-high school training	38 232	23.4	88 837	54.5	23 482	14.4	12 552	7.7
Completed college	17 308	18.6	49 650	53.4	17 507	18.8	8447	9.1
Completed graduate school	17 501	18.0	53 672	55.0	18 948	19.4	7393	7.6
								
*Daily cigarette use (current or former)* (%)								
Never	52 199	29.8	97 856	55.9	19 103	10.9	5981	3.4
Former	49 726	20.6	125 703	52.1	43 831	18.2	21 819	9.1
Current	14 821	21.7	33 980	49.8	10 747	15.7	8717	12.8
								
*Usual daily physical activity*								
Sit during the day/little walking	10 151	26.1	20 956	53.8	5072	13.0	2758	7.1
Sit during the day/walk a fair amount	36 178	22.8	87 295	55.0	23 823	15.0	11 338	7.2
Stand/walk a lot – no lifting	44 768	24.1	97 928	52.7	28 842	15.5	14 187	7.6
Lift/carry light loads, stairs, hills	20 080	23.8	43 627	51.6	13 953	16.5	6892	8.2
Do heavy work/carry loads	4128	29.1	6660	47.0	1955	13.8	1437	10.1
								
*Physical activity (leisure) frequency*								
Never	8771	40.1	9454	43.3	2070	9.5	1556	7.1
Rarely	19 302	28.9	34 540	51.7	7613	11.4	5310	8.0
1–3 times per month	15 299	22.9	37 126	55.5	9221	13.8	5223	7.8
1–2 times per week	23 055	21.7	58 530	55.2	16 491	15.6	7952	7.5
3–4 times per week	28 304	21.5	71 987	54.6	22 124	16.8	9504	7.2
5 or more times per week	22 600	23.9	47 856	50.6	16 818	17.8	7375	7.8

a Categories may not add up to 492 960 persons owing to missing data.

b Median (interquartile range).

**Table 2:** Age-adjusted risk ratios and incidence rates for head and neck cancer by alcohol use

		**Age-adjusted**			
**Total alcohol consumption**	**Cases**	**HR**	**95% CI**	**Incidence rates^a^ per 100 000 person-years**	**95% CI**
*Men*					
0 drinks per day	165	1.77	1.45–2.16	61.3	51.9–70.7
<1 drink per day	226	1.00	(ref)	34.4	30.0–39.0
1–3 drinks per day	110	1.29	1.03–2.82	44.5	36.1–52.8
>3 drinks per day	110	2.25	1.79–2.82	77.6	63.1–92.1
					
*Women*					
0 drinks per day	54	1.39	0.98–1.98	20.4	14.9–25.8
<1 drink per day	75	1.00	(ref)	14.7	11.3–18.0
1–3 drinks per day	36	2.69	1.81–4.00	39.6	26.6–52.5
>3 drinks per day	18	5.16	3.08–8.63	75.3	40.5–110.1

95% CI=95% confidence interval; HR=hazard ratio.

a Incidence rates by alcohol category and gender were applied to the entire AARP population.

**Table 3:** Adjusted HR and 95% CI for alcohol intake and head and neck cancer

	**Head and neck cancer^a^**					
	**Men**			**Women**		
	**Cases**	**HR**	**95% CI**	**Cases**	**HR**	**95% CI**
*Total alcohol*						
0 drinks per day	165	1.68	1.37–2.06	54	1.46	1.02–2.08
<1 drink per day	226	1.00	(ref)	75	1.00	(ref)
1–3 drinks per day	110	1.23	0.98–1.55	36	1.99	1.33–2.99
>3 drinks per day	110	1.48	1.15–1.90	18	2.52	1.46–4.35
*P*-value for trend^b^		0.001			0.0002	
						
*Wine* c						
0 drinks per day	318	1.34	1.10–1.63	91	1.58	1.12–2.25
<1 drink per day	252	1.00	(ref)	74	1.00	(ref)
1–3 drinks per day	40	1.16	0.83–1.62	18	1.97	1.17–3.31
>3 drinks per day	1	0.43	0.06–3.05	0		
*P*-value for trend^b^		0.872			0.332	
						
*Beer* d						
0 drinks per day	230	1.18	0.96–1.45	107	0.85	0.60–1.19
<1 drink per day	286	1.00	(ref)	67	1.00	(ref)
1–3 drinks per day	41	1.24	0.89–1.72	3	0.85	0.27–2.73
>3 drinks per day	54	1.61	1.16–2.22	6	3.40	1.40–8.24
*P*-value for trend^b^		0.002			0.084	
						
*Liquor* e						
0 drinks per day	294	1.28	1.03–1.58	92	1.16	0.80–1.66
<1 drink per day	195	1.00	(ref)	62	1.00	(ref)
1–3 drinks per day	61	1.53	1.14–2.04	17	1.86	1.08–3.19
>3 drinks per day	61	1.85	1.37–2.50	12	2.25	1.19–4.26
*P*-value for trend^b^		<.0001			0.003	

95% CI=95% confidence interval; HR=hazard ratio.

a Adjusted for gender, age at entry into cohort, body mass index, education, smoke-quit-dose, vigorous physical activity, usual activity throughout the day, fruit intake, vegetable intake, and total energy.

b The test for trend was restricted to alcohol drinkers and used a linear variable where each subject was assigned the median intake value for their category.

c Additionally adjusted for categories of beer and liquor.

d Additionally adjusted for categories of liquor and wine.

e Additionally adjusted for categories of wine and beer.

**Table 4:** Adjusted HR and 95% CI for head and neck cancer subtypes by gender

	**Oral cavity^a^**						**Oropharynx and hypopharynx^a^**						**Larynx^a^**					
**Cancer Sub-site**	**Men**			**Women**			**Men**			**Women**			**Men**			**Women**		
**Gender**	**Cases**	**HR**	**95% CI**	**Cases**	**HR**	**95% CI**	**Cases**	**HR**	**95% CI**	**Cases**	**HR**	**95% CI**	**Cases**	**HR**	**95% CI**	**Cases**	**HR**	**95% CI**
*Total alcohol*																		
0 drinks per day	57	1.43	1.03–2.00	24	1.24	0.74–2.10	37	2.83	1.74–4.61	9	1.50	0.63–3.58	59	1.55	1.11–2.16	15	1.39	0.71–2.74
<1 drink per day	92	1.00	(ref)	38	1.00	(ref)	30	1.00	(ref)	13	1.00	(ref)	88	1.00	(ref)	21	1.00	(ref)
1–3 drinks per day	44	1.22	0.85–1.76	15	1.74	0.95–3.20	18	1.53	0.85–2.76	10	3.24	1.40–7.52	41	1.18	0.82–1.72	9	1.56	0.71–3.43
>3 drinks per day	43	1.52	1.01–2.27	9	2.81	1.29–6.11	24	2.32	1.29–4.18	2	1.97	0.42–9.31	41	1.37	0.91–2.05	6	2.15	0.82–5.65
*P*-value for trend^b^		0.062			0.013			0.003			0.132			0.095			0.042	
																		
*Wine* c																		
0 drinks per day	120	1.46	1.07–1.99	40	1.43	0.86–2.39	60	1.39	0.87–2.24	17	1.87	0.83–4.23	121	1.27	0.93–1.74	26	1.34	0.70–2.56
<1 drink per day	102	1.00	(ref)	37	1.00	(ref)	38	1.00	(ref)	13	1.00	(ref)	95	1.00	(ref)	21	1.00	(ref)
1–3 drinks per day	14	0.97	0.55–1.70	9	2.07	0.99–4.34	10	1.98	0.98–4.00	4	2.28	0.74–7.09	13	1.03	0.58–1.84	4	1.56	0.53–4.59
>3 drinks per day	0			0			1	2.59	0.35–19.05	0			0			0		
*P*-value for trend^b^		0.401			0.451			0.093			0.400			0.530			0.789	
																		
*Beer* d																		
0 drinks per day	79	0.95	0.68–1.34	49	0.83	0.50–1.35	52	2.52	1.53–4.17	17	0.49	0.22–1.07	83	1.00	0.72–1.39	34	1.39	0.71–2.71
<1 drink per day	117	1.00	(ref)	32	1.00	(ref)	36	1.00	(ref)	16	1.00	(ref)	115	1.00	(ref)	14	1.00	(ref)
1–3 drinks per day	17	1.30	0.78–2.18	1	0.67	0.09–4.97	8	1.83	0.85–3.97	1	1.14	0.15–8.86	14	1.01	0.58–1.77	1	1.17	0.15–9.01
>3 drinks per day	23	1.78	1.08–2.94	4	5.96	1.94–18.30	13	3.12	1.54–6.31	0			17	1.14	0.65–1.98	2	3.76	0.78–18.23
*P*-value for trend^b^		0.016			0.092			0.0003			0.517			0.772			0.122	
																		
*Liquor* e																		
0 drinks per day	109	1.20	0.86–1.68	42	1.04	0.62–1.75	53	1.16	0.68–1.96	19	2.23	0.90–5.51	114	1.49	1.06–2.10	24	0.87	0.40–1.68
<1 drink per day	81	1.00	(ref)	32	1.00	(ref)	29	1.00	(ref)	8	1.00	(ref)	71	1.00	(ref)	20	1.00	(ref)
1–3 drinks per day	25	1.56	0.99–2.45	6	1.35	0.56–3.26	14	2.36	1.24–4.49	5	4.28	1.38–13.27	19	1.27	0.77–2.12	4	1.17	0.40–3.44
>3 drinks per day	21	1.65	1.00–2.72	6	2.45	0.99–6.05	13	2.52	1.28–4.99	2	3.28	0.66–16.27	25	1.99	1.24–3.21	3	1.31	0.38–4.57
*P*-value for trend^b^		0.068			0.072			0.009			0.135			0.001			0.298	

95% CI=95% confidence interval; HR=hazard ratio.

a Adjusted for gender, age at entry into cohort, body mass index, education, smoke-quit-dose, vigorous physical activity, activity throughout the day, fruit intake, vegetable intake, and total energy.

b The test for trend was restricted to alcohol drinkers and used a linear variable where each subject was assigned the median intake value for their category.

c Additionally adjusted for categories of beer and liquor.

d Additionally adjusted for categories of liquor and wine.

e Additionally adjusted for categories of wine and beer.
